# Assessment of Oxidative Stress and Antioxidant Status in Allergic Rhinitis

**DOI:** 10.3390/biomedicines14010189

**Published:** 2026-01-15

**Authors:** Ahmet Burak Gürpınar, Selen Karaoğlanoğlu

**Affiliations:** 1 Department of Medical Biochemistry, Faculty of Medicine, Tokat Gaziosmanpaşa University, 60030 Tokat, Türkiye; abgurp@yahoo.com; 2 Department of Pulmonology, Faculty of Medicine, Ordu University, 52200 Ordu, Türkiye

**Keywords:** allergic rhinitis, oxidative stress, thiol–disulfide homeostasis, total antioxidant status, total oxidant status

## Abstract

**Background:** Allergic rhinitis (AR) is a chronic immunoglobulin E (IgE)-mediated inflammatory disorder triggered by aeroallergens. Oxidative stress (OS) is increasingly recognized as a key factor in AR pathophysiology. This study aimed to investigate dynamic thiol–disulfide homeostasis (TDH) and OS markers in AR patients compared to healthy controls. **Methods:** Sixty-two participants (31 AR patients, 31 controls) were enrolled. Hematological and biochemical parameters were measured. OS markers including total thiol (TT), native thiol (NT), disulfide, total antioxidant status (TAS), total oxidant status (TOS), and oxidative stress index (OSI) were assessed. Correlations between OS markers and laboratory parameters were analyzed. Receiver operating characteristic (ROC) analysis evaluated the diagnostic performance of OS markers. **Results:** TT and NT levels were significantly lower in AR patients, whereas disulfide, disulfide/NT and disulfide/TT ratios, TOS and OSI were significantly higher. TAS levels were slightly lower in AR patients. TT and NT correlated positively with eosinophil counts and negatively with monocyte, platelet, AST, and creatinine levels. ROC analysis indicated strong diagnostic potential: TT (AUC = 0.749, cutoff 415 µmol/L, sensitivity 90%, specificity 61%), NT (AUC = 0.786, cutoff 373.2 µmol/L, sensitivity 90%, specificity 71%), and disulfide (AUC = 0.690, cutoff 20 µmol/L, sensitivity 74%, specificity 61%). **Conclusions:** AR patients exhibit disrupted TDH and elevated OS. These markers may serve as sensitive indicators of oxidative imbalance, offering potential diagnostic and therapeutic insights into AR management.

## 1. Introduction

Allergic rhinitis (AR) has become a significant global health concern over the past two decades, leading to substantial reductions in quality of life and increased healthcare expenditures. AR is characterized by chronic upper airway inflammation triggered by an IgE-mediated T helper 2 (Th2) response. Allergen exposure, recognized by dendritic cells, promotes Th2 cell differentiation and increases the release of cytokines such as IL-4, IL-5, and IL-13, which in turn elevate IgE production and lead to mast cell and eosinophil activation. This immune response triggers both the release of proinflammatory mediators and reactive oxygen species (ROS) production via eosinophils in the mucosal epithelium. ROS damage cellular lipids, proteins, and DNA through oxidative stress (OS) and particularly disrupt airway epithelial barrier integrity; this increases epithelial permeability, further intensifying allergen penetration and the inflammatory response [1,2].

Thiol disulfide homeostasis (TDH) is a critical indicator of cellular redox balance and is determined by the ratio of low molecular weight thiols (e.g., glutathione, free –SH groups) to disulfide bonds. When OS increases, thiol groups oxidize and convert to disulfide bonds; this redox shift can affect protein structure and signal transduction. Increased oxidative load in AR leads to epithelial oxidative damage and depletion of antioxidant defenses; this process causes a shift in the thiol/disulfide balance toward disulfides. Clinical studies have observed impaired TDH in AR patients, including decreased total thiol (TT) and native thiol (NT) levels and increased disulfide levels, which is considered a reflection of systemic OS [3,4]

In this context, the interaction between the Th2-driven immune response and epithelial ROS production not only increases the severity of inflammation but also disrupts redox balance, affecting TDH. This mechanistic link supports approaching AR pathogenesis from anOS perspective and evaluating thiol–disulfide balance as both a biomarker and a potential therapeutic target [5,6,7,8].

## 2. Materials and Methods

### 2.1. Study Design and Population

This prospective observational study was conducted at the Chest Diseases and Allergy–Immunology outpatient clinics of Ordu University Training and Research Hospital, a tertiary care center, between May and November 2023. The study included 31 patients diagnosed with AR (20 females and 11 males) and a control group of healthy volunteers.

The control group consisted of 31, age and sex comparable healthy individuals recruited from individuals attending routine outpatient visits for non-inflammatory, non-allergic conditions. Control participants had no history of AR, asthma, or other atopic or chronic inflammatory diseases. Although individual matching was not performed, the AR and control groups were comparable in terms of age and sex. Demographic characteristics of both groups are presented in Section 3.

All AR diagnoses were established according to the ARand its Impact on Asthma (ARIA) criteria by an allergy and immunology specialist. Written informed consent was obtained from all participants prior to enrollment.

### 2.2. Ethical Considerations

The study protocol was approved by the Ethics Committee of Non-Interventional Clinical Research of Ordu University (Decision No: 2023/92, Date: 31 March 2023) and was conducted in accordance with the principles of the Declaration of Helsinki.

### 2.3. Inclusion and Exclusion Criteria

Patients were included if they had a confirmed diagnosis of AR according to ARIA criteria, were in a clinically stable phase without acute exacerbation, had not received inhaled or systemic corticosteroids, antihistamines, immunotherapy, or antioxidant supplementation within the preceding three months, and provided written informed consent.

Exclusion criteria included refusal to participate, unconfirmed AR diagnosis, presence of acute infection or exacerbation, chronic systemic inflammatory, hepatic, renal, or malignant disease, and current use of medications known to affect OS parameters.

The same exclusion criteria were applied to the control group.

### 2.4. Sample Collection and Laboratory Analysis

Venous blood samples were collected from all participants from the antecubital vein in the morning after an overnight fasting period.

For hematological analyses, approximately 2 mL of blood was collected into EDTA-K2–containing tubes. Samples were gently inverted and analyzed within two hours using an automated hematology analyzer (Sysmex XN-1000, Sysmex Corporation, Kobe, Japan).

For biochemical analyses, approximately 8 mL of blood was collected into serum separator tubes without anticoagulant. Samples were allowed to clot at room temperature for approximately 20 min and then centrifuged at 3000 rpm for 10 min. Serum levels of albumin, C-reactive protein (CRP), urea, creatinine, alanine aminotransferase (ALT), aspartate aminotransferase (AST), and lactate dehydrogenase (LDH) were measured using an automated biochemistry analyzer (Cobas 8000 c702, Roche Diagnostics, Mannheim, Germany).

All laboratory measurements were performed at the Central Biochemistry Laboratory of Ordu University Training and Research Hospital by experienced laboratory personnel blinded to the clinical group allocation.

### 2.5. Assessment of Oxidative Stress and Thiol-Disulfide Homeostasis

To evaluate OS and antioxidant capacity, serum samples were aliquoted and stored at −80 °C until batch analysis.

Total antioxidant status (TAS), total oxidant status (TOS), NT, and TT levels were measured using fully automated spectrophotometric methods with commercially available kits (Rel Assay Diagnostics, Gaziantep, Türkiye) [9]. The inter- and intra-assay coefficients of variation were 2.8% and 3.3% for TAS, and 3.2% and 3.9% for TOS, respectively.

Disulfide levels were calculated as half of the difference between TT and NT concentrations. To reduce analytical variability, measurements were performed in duplicate, and mean values were used for statistical analysis.

### 2.6. Statistical Analysis

Sample size estimation was performed using G-Power software (version 3.1.2). Assuming a large effect size (Cohen’s d = 0.80), an alpha level of 0.05, and a power of 80%, a minimum of 30 participants per group was estimated.

Statistical analyses were conducted using MedCalc software (version 20.009; MedCalc Software Ltd., Ostend, Belgium). Normality of data distribution was assessed using the Shapiro–Wilk test. Continuous variables were expressed as mean ± standard deviation or median (interquartile range), as appropriate. Categorical variables were presented as frequencies and percentages.

Comparisons between groups were performed using the Independent Samples t-test or Mann–Whitney U test, depending on data distribution. Categorical variables were compared using the chi-square test. Correlation analyses were performed using Pearson or Spearman correlation coefficients. Receiver operating characteristic (ROC) curve analysis was used to evaluate the diagnostic performance of OS parameters. A *p*-value < 0.05 was considered statistically significant.

## 3. Results

### 3.1. Study Population and Baseline Characteristics

A total of 62 participants were included in this study, comprising 31 patients with AR (20 females and 11 males) and 31 healthy controls (18 females and 13 males). There were no significant differences between the groups in terms of gender, age, or body mass index (BMI) (*p* = 0.605, *p* = 0.783, and *p* = 0.878, respectively).

### 3.2. Hematological and Biochemical Findings

In the AR group, monocyte, platelet, and hemoglobin levels were significantly higher compared to the control group (*p* = 0.001, *p* = 0.004, and *p* = 0.023, respectively). Conversely, lymphocyte and eosinophil counts were significantly lower than those in the control group (*p* = 0.046 and *p* < 0.001, respectively).

There were no statistically significant differences in WBC or neutrophil counts (*p* = 0.072 and *p* = 0.364, respectively). Among biochemical parameters, albumin and urea levels were higher in the AR group, though the differences were not statistically significant (*p* = 0.068 and *p* = 0.106, respectively), whereas CRP levels were lower (*p* = 0.083). ALT levels were significantly lower in the AR group, while AST and creatinine levels were significantly higher (*p* = 0.026, *p* < 0.0001, and *p* < 0.0001, respectively) (Table 1).

### 3.3. Clinical Characteristics of AR Patients

Among the AR patients, 19.4% had concomitant asthma, most of which were classified as mild (3.2%) or severe (16.1%). Polysensitization (≥2 allergens) was observed in 71.0%, while monosensitization was present in 16.1% (Table 2).

**Figure 1:**
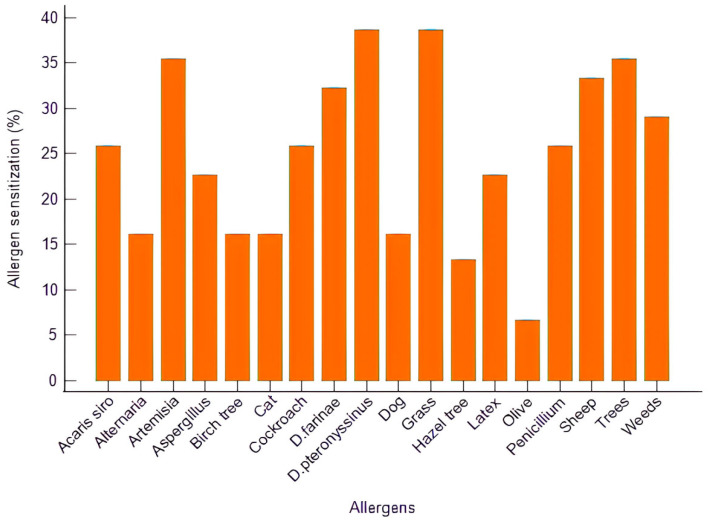
Distribution of allergen sensitization of the AR patients.

The most frequent sensitizations involved grass pollen (38.7%), *Dermatophagoides pteronyssinus* (38.7%), tree pollen (35.5%), *Artemisia* (35.5%), and sheep dander (33.3%), followed by *D. farinae* (32.3%) and weed pollens (29.0%). Sensitization to molds such as *Aspergillus*, *Penicillium*, and *Alternaria* was also detected (Figure 1).

Subgroup analyses demonstrated no statistically significant differences in OS parameters between AR patients with and without asthma (*p* > 0.05). Similarly, no significant differences in OS parameters were observed between monosensitized and polysensitized patients (*p* > 0.05). Detailed subgroup comparison results are provided in (Table 3, Table 4 and Table 5).

### 3.4. TDH and Other OS Markers

**Figure 2:**
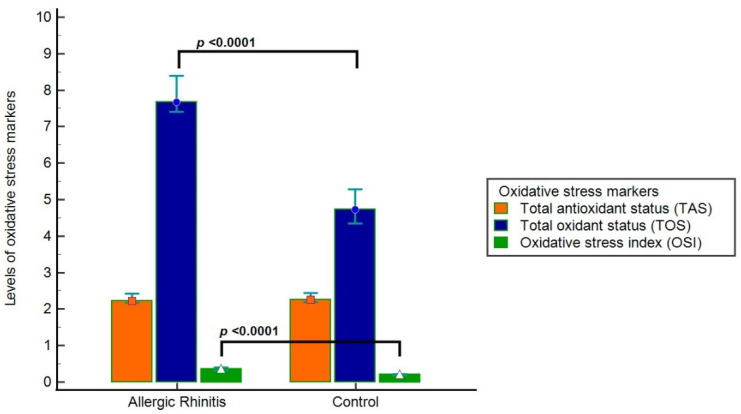
Comparison results on TAS, TOS and OSI in the groups. Groups are indicated as median and 95% CI (confidence interval).

Compared with controls, patients with AR exhibited significantly lower TT, NT, and NT/TT ratios (*p* = 0.001, *p* < 0.001, and *p* = 0.001, respectively). Disulfide levels, disulfide/NT ratio, and disulfide/TT ratio were significantly higher in the AR group (*p* = 0.012, *p* = 0.001, and *p* = 0.001, respectively). In addition, TOS and OSI were significantly elevated in the AR group (*p* < 0.0001 for both), while TAS levels were lower without reaching statistical significance (*p* = 0.363) (Table 6, Figure 2).

### 3.5. Correlation Analyses

TT levels positively correlated with eosinophils (r = 0.259, *p* = 0.042) and negatively correlated with creatinine (r = −0.255, *p* = 0.045, respectively).

NT levels positively correlated with eosinophils (r = 0.290, *p* = 0.022) and negatively correlated with monocytes, platelets, creatinine, and AST (r = −0.305, *p* = 0.016; r = −0.278, *p* = 0.029; r = −0.269, *p* = 0.034; r = −0.308, *p* = 0.015, respectively).

Disulfide levels positively correlated with monocytes (r = 0.343, *p* = 0.006).

TAS levels showed no significant correlations. TOS levels positively correlated with monocytes, platelets, hemoglobin, creatinine, and AST (*p* ≤ 0.003 for all), and negatively correlated with eosinophils and ALT (*p* ≤ 0.010). OSI demonstrated similar correlations, positively correlating with monocytes, platelets, hemoglobin, albumin, creatinine, and AST (*p* ≤ 0.003), and negatively with eosinophils and ALT (*p* ≤ 0.008) (Table 7).

### 3.6. Diagnostic Performance of OS Parameters

In the ROC analysis performed to evaluate the diagnostic performance of OS parameters in AR, the optimal cutoff value for TT was determined as 415, with 90% sensitivity and 61% specificity. For NT, the optimal cutoff value was 373.2, corresponding to 90% sensitivity and 71% specificity. The optimal cutoff value for disulfide was identified as 20, with 74% sensitivity and 61% specificity (Table 8).

**Figure 3:**
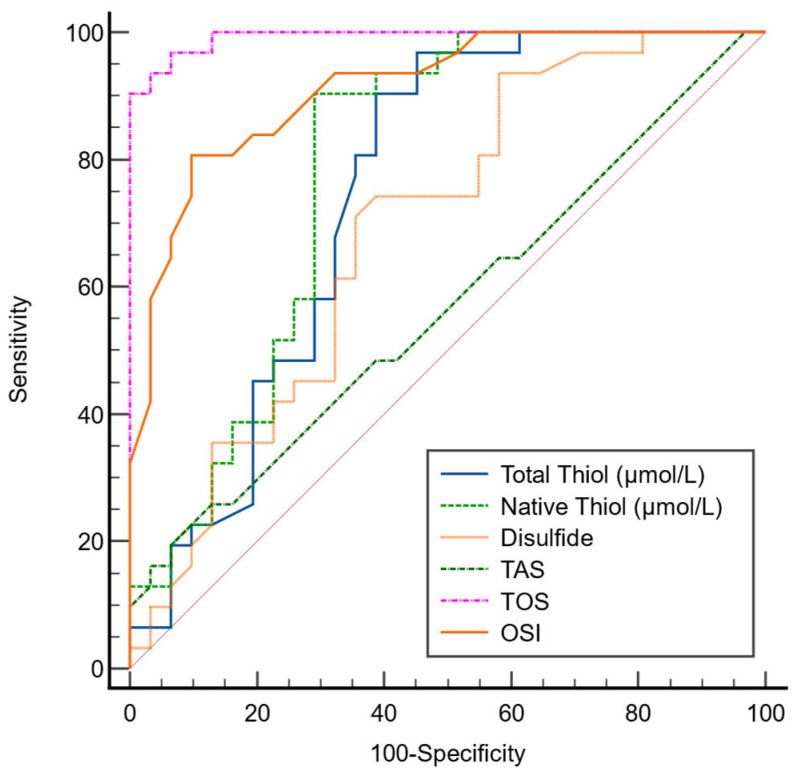
ROC curve comparison of OS markers for AR. The diagonal line (pink dotted line) represents fifty percent of the area in the ROC analysis graph.

Among the OS parameters, the AUC values for TT, NT, and disulfide were found to be 0.749, 0.786, and 0.690, respectively (Figure 3).

## 4. Discussion

AR is characterized by chronic Th2-mediated inflammation of the upper airway and is increasingly recognized as a condition involving systemic redox dysregulation. Experimental and clinical studies indicate that OS amplifies inflammation by promoting epithelial barrier dysfunction, allergen penetrance, and immune cell activation through redox-sensitive signaling pathways. Within this context, TDH has emerged as a sensitive surrogate marker of redox status, reflecting the balance between antioxidant thiols and oxidized disulfides.

A surprising finding in the current study was the significantly reduced eosinophil count in patients with AR relative to control subjects that may counter the classical association between allergic diseases and eosinophilia. But peripheral blood eosinophil levels do not represent local tissue eosinophilic inflammation, specifically in patients with stable disease or during nonspecific acute symptomatic exacerbations. Many studies showed eosinophilic infiltration in AR predominantly to the nasal mucosal level and circulating eosinophil counts may be intact or decreased owing to tissue migration. In the absence of symptom severity scoring and the cross-sectional design of the study may also have inhibited estimation of disease activity at the time of sampling. Thus, the lower eosinophil levels seen in our population could be due to the disease phase, timing of blood sampling, or redistribution of eosinophils from peripheral circulation to inflamed nasal tissues rather than lack of eosinophilic inflammation [10,11,12].

The decrease in NT and TT levels seen followed by increased levels of disulfides, combined with the significant increase in disulfide concentrations indicates elevated consumption of thiol groups by the body in response to excessive ROS production in AR. Thiols are important constituents of the antioxidant defense system as they consume ROS and preserve redox sensitive signaling pathways. However, under sustained inflammatory activation, like that found in AR, ROS mediated oxidation occurs due to the generation of too many ROS of thiol groups, and allows disulfide bond generation, thus the TDH balance is shifted into an oxidized state. This change potentially leads to impaired epithelial barrier stability, enhanced allergen penetration, and maintenance of local inflammatory responses. Thus, the revised TDH profile observed in our cohort is probably due to augmented oxidative burden and reduced antioxidant buffering capacity in AR [13,14].

A previous prospective observational study including 35 patients with persistent AR demonstrated that treatment significantly increased NT and NT/TT ratios while decreasing disulfide and disulfide related ratios, indicating a reduction in OS following therapy despite ongoing allergen exposure [15]. These results align with our findings, which showed that untreated AR patients had markedly lower TT and NT levels and higher disulfide and OSI values compared to healthy controls. Together, these data suggest that AR is characterized by an imbalance in TDH, reflecting elevated oxidative burden, and that this imbalance may be reversible with effective treatment. Both studies used an automated spectrophotometric method to assess TDH, supporting methodological consistency. The observed increase in thiol levels following treatment in the previous study likely reflects restoration of antioxidant capacity via decreased ROS generation and enhanced thiol-dependent redox buffering. In contrast, our results represent the pre-treatment state of oxidative imbalance, emphasizing the potential role of TDH parameters not only in disease pathogenesis but also as sensitive markers for monitoring therapeutic response in AR.

Similarly, in a study including 32 patients with seasonal allergic rhinitis (SAR), TDH parameters were evaluated both during symptomatic exacerbations and asymptomatic periods. The authors found that during exacerbations, disulfide levels and related ratios were significantly elevated, whereas NT levels were significantly reduced compared with asymptomatic periods, indicating increased OS during active disease. Interestingly, NT and TT levels in asymptomatic SAR patients were comparable to those of healthy controls, suggesting a reversible oxidative imbalance linked to allergen exposure [8]. Our findings are consistent with these results, as we also observed a significant decrease in TT and NT levels and an increase in disulfide, TOS, and OSI in AR patients compared to healthy controls, reflecting enhanced OS. These parallels across both seasonal and persistent forms of AR support the concept that OS plays a key role in disease activity and symptom fluctuation. Taken together, they highlight TDH as a sensitive indicator of redox imbalance that may fluctuate with allergen load and clinical status, offering potential utility in both disease monitoring and treatment optimization.

In a recent study investigating the relationship between SAR and TDH together with trace element status, significantly lower serum copper, NT, and reduced thiol levels, along with a decreased thiol oxidation–reduction ratio, were reported in the AR group compared to healthy controls, indicating increased OS and impaired antioxidant defense mechanisms (*p* < 0.05) [7]. In contrast to these findings, our study demonstrated that although TT, NT, and disulfide levels were altered in patients with AR, the differences did not reach statistical significance when compared with the control group. This discrepancy may be attributed to variations in disease phenotype (seasonal versus perennial), environmental allergen exposure, and sample size. Furthermore, the inclusion of patients with both mono and polysensitization patterns in our cohort may have contributed to the absence of significant differences. Nevertheless, consistent with the literature, our data support the notion that OS and TDH are implicated in the pathophysiology of AR, even if the degree of imbalance varies according to clinical and environmental factors.

In our analysis, while TOS and OSI levels were raised significantly in AR patients, there was no difference between groups in TAS levels. This observation may indicate a compensatory upregulation of antioxidant mechanisms in response to increased oxidative burden, resulting in relatively preserved total antioxidant capacity despite ongoing OS. Additionally, TAS is an aggregate measure influenced by various endogenous and exogenous factors, which may result in reduced sensitivity to identify subtle redox alterations in small groups. By contrast, TOS and OSI provide a more straightforward representation of oxidative load, a potential reason for their higher discriminatory ability in the current research [5,16].

When compared to the study by Moon and colleagues [6], which investigated 226 AR students and focused on TAS and TOS levels relative to symptom duration and severity, some similarities and differences emerge. Both studies highlight the relevance of OS in AR. While their study reported higher TAS levels in individuals with symptom duration of more than six months, suggesting a compensatory increase in antioxidant activity with chronicity, our study did not find a statistically significant difference in TAS between AR patients and controls. However, we observed a significant increase in TOS and OSI levels, supporting the presence of OS in AR. This discrepancy may be due to differences in study populations, including age, sample size, and disease chronicity, as well as the parameters used to assess OS.

There were no statistically significant differences when accounting for asthma comorbidity or sensitization pattern. This observation is consistent with past data showing that upper airway inflammation in AR might actually lead to enough oxidative and endothelial changes independent of lower airway involvement. In a prospective cross-sectional study among adolescents with AR, nasal OS markers and endothelial injury indicators were similarly elevated in patients with and without asthma, suggesting that the presence of asthma does not necessarily confer an additional oxidative burden. Importantly, the authors found comparable increases in TOS and OSI across both subgroups, reinforcing the concept that AR itself is capable of driving significant redox imbalance [17].

Mechanistically, AR is characterized by ongoing Th2-driven inflammation and oxidative damage to the upper airway epithelium, processes sufficient to independently generate ROS and thiol oxidation, without the presence of comorbidity. Despite the prevalent systemic inflammatory load associated with asthma, the absence of meaningful differences in OS parameters within our cohort was striking. This may indicate that upper airway inflammation alone can saturate redox sensitive pathways, blurring potentially additive effects of asthma [18]. Similarly, no significant differences between monosensitized and polysensitized patients suggest that OS may be triggered by allergic inflammation rather than the number of sensitizing allergens. This observation is in line with previous reports indicating that OS markers reflect inflammatory activation rather than allergen load [19]. We expect this to be consistent with earlier findings that OS markers are reflective of inflammatory activation rather than allergen load. Yet, it should be considered that the small sample size and subgroup distribution might have compromised the statistical power toward identifying fine differences between these subgroups. Taken together, these findings imply that thiol–disulfide imbalance in AR may occur early in the disease process and remain relatively stable across different clinical phenotypes. Larger studies with stratification by disease severity, asthma control, and quantitative allergen exposure are warranted to further clarify the relationship between OS, sensitization patterns, and comorbid asthma.

The correlations between TDH markers and the inflammatory cell indicators observed here may be due to the interplay of redox abnormality and systemic immune activation under AR circumstances. However, these associations should not be construed as causal relationships and must be interpreted cautiously due to the limited sample size. Similar links on OS markers and inflammatory parameters have been established for allergic and inflammatory symptoms, which supports the biological plausibility of results obtained in our study [3,6,20]. Future studies specifically designed to investigate these relationships are warranted.

The ROC analysis revealed that TT and NT levels exhibited moderate diagnostic performance in distinguishing AR patients from healthy controls. These findings suggest that TDH parameters may serve as supportive biomarkers reflecting oxidative imbalance in AR rather than standalone diagnostic tools. Given the modest sample size, the diagnostic accuracy estimates should be interpreted with caution, and external validation in larger cohorts is required before clinical implementation. Nonetheless, these results highlight the potential utility of thiol-based markers as adjunctive indicators of OS related inflammation in AR [21].

Our study has several limitations. First, the relatively small sample size and single-center design may limit the generalizability of our findings and may have increased the risk of both type I and type II statistical errors. Second, potential seasonal and environmental confounders, which are known to influence AR activity and OS parameters, were not systematically controlled. Third, clinical symptom severity scores were not recorded; therefore, correlations between TDH parameters and disease severity could not be evaluated. In addition, allergen load or individual exposure levels were not assessed, which may have contributed to inter-individual variability in OS markers. Finally, the cross-sectional design without longitudinal follow-up or repeated measurements precludes conclusions regarding temporal changes in redox balance or its relationship with disease progression and treatment response. Future multicenter studies with larger cohorts, standardized environmental assessments, and longitudinal designs are warranted to confirm and extend our findings.

## 5. Conclusions

In summary, AR is associated with significant disruption of TDH, characterized by decreased thiol levels and increased disulfide formation, reflecting an enhanced systemic oxidative burden. These alterations occurred independently of asthma comorbidity and sensitization pattern, suggesting that OS represents a fundamental feature of AR pathophysiology. Although TDH parameters demonstrated only moderate diagnostic performance, they may serve as supportive biomarkers for evaluating oxidative burden or monitoring treatment response. Overall, the present findings add to the evidence linking OS to allergic airway inflammation and highlight TDH as a promising target for future mechanistic and translational studies.

## Figures and Tables

**Table 1:** Demographic features and comparison results on laboratory parameters in the groups.

		Groups				*p*-Value
		Allergic Rhinitis		Control		
		*n* = 31		*n* = 31		
Demographic findings						
Gender	Female, *n* (%)	20	(64.5)	18	(58.1)	0.605
	Male, *n* (%)	11	(35.5)	13	(41.9)	
Age (years), mean (SD)		29.3	(9)	29.8	(6.3)	0.783
BMI (kg/m^2^), mean (SD)		23.7	(3.8)	23.5	(4.4)	0.878
Hematological results						
WBC (10^3^/L), median (25 p–75 p)		7.0	(5.2–8.1)	7.6	(6.2–8.7)	0.072
Neutrophil (10^3^/L), median (25 p–75 p)		3.1	(2.6–4.7)	3.7	(2.9–4.2)	0.364
Lymphocyte (10^3^/L), median (25 p–75 p)		2.0	(1.7–2.7)	2.5	(2.1–2.9)	0.046 **
Monocyte (10^3^/L), median (25 p–75 p)		0.5	(0.4–0.6)	0.4	(0.3–0.5)	0.001 **
Eosinophil (10^3^/L), median (25 p–75 p)		0.2	(0.1–0.3)	0.3	(0.2–0.4)	0.000 **
Platelet (10^3^/L), median (25 p–75 p)		260	(231–306)	225	(205–261)	0.004 **
Hemoglobin (g/dL), median (25 p–75 p)		13.0	(12.6–14.7)	12.6	(11.2–13.3)	0.023 **
Biochemical results						
Albumin (g/dL), mean (SD)		4.6	(0.3)	4.5	(0.3)	0.068
CRP (mg/dL), median (25 p–75 p)		0.1	(0.06–0.14)	0.2	(0.08–0.34)	0.074
Urea (mg/dL), mean (SD)		20	(8.7)	17	(4)	0.106
Creatinine (mg/dL), mean (SD)		0.7	(0.1)	0.5	(0.1)	<0.0001 *
ALT (IU/L), median (25 p–75 p)		17	(11–19)	19	(16–21)	0.026 **
AST (IU/L), median (25 p–75 p)		16	(13–20)	10	(8–11)	<0.0001 **

* Significant difference at <0.05 level according independent t-test, Means and Standard deviations (SD) are presented ** Significant difference at <0.05 level according to Mann–Whitney U test, Medians are presented and 25 p–75 p are shown in parentheses.

**Table 2:** Clinical characteristics of AR patients.

		Allergic Rhinitis	
		*n*	%
Asthma	Yes	6	19.4
	No	25	80.6
Mild asthma	Yes	1	3.2
	No	30	96.8
Moderate asthma	Yes	0	0.0
	No	31	100.0
Severe asthma	Yes	5	16.1
	No	26	83.9
Atopy	Yes	27	87.1
	No	4	12.9
Monosensitization	Yes	5	16.1
	No	26	83.9
Polysensitization	Yes	22	71.0
	No	9	29.0

**Table 3:** Comparison of OS parameters in AR patients with and without asthma.

Oxidative Stress Markers	Allergic Rhinitis				*p*-Value
	Asthma = Yes		Asthma = No		
	*n* = 6		*n* = 25		
TT (µmol/L), median (25 p–75 p)	409	(383–413)	385	(362–410)	0.177
NT (µmol/L), mean (SD)	352	(18)	339	(33)	0.370
Disulfide, mean (SD)	24	(4)	24	(5)	0.790
Disulfide/Native Thiol (%), median (25 p–75 p)	6.8	(5.6–8.3)	6.6	(5.4–8.4)	0.635
Disulfide/Total Thiol (%), median (25 p–75 p)	5.9	(5–7.1)	5.8	(4.8–7.2)	0.881
NT/TT (%), median (25 p–75 p)	88.1	(86–90)	88.4	(86–90)	0.881
TAS, mean (SD)	2.3	(0.4)	2.3	(0.7)	0.857
TOS, mean (SD)	7.7	(0.6)	7.9	(1)	0.598
OSI, mean (SD)	0.34	(0.06)	0.39	(0.13)	0.438

Means and Standard deviations (SD) are presented Medians are presented and 25 p–75 p are shown in parentheses.

**Table 4:** Comparison of OS markers by monosensitization status in patients with AR.

Oxidative Stress Markers	Allergic Rhinitis				*p*-Value
	Monosensitization = Yes		Monosensitization = No		
	*n* = 5		*n* = 26		
TT (µmol/L), median (25 p–75 p)	385	(371–410)	400	(369–410)	0.872
NT (µmol/L), mean (SD)	341	(25)	342	(32)	0.938
Disulfide, mean (SD)	23	(3)	24	(5)	0.735
Disulfide/Native Thiol (%), median (25 p–75 p)	6.6	(2.9–7.4)	6.7	(5.3–8.4)	0.914
Disulfide/Total Thiol (%), median (25 p–75 p)	5.8	(5.3–6.4)	5.9	(4.8–7.2)	0.915
NT/TT (%), median (25 p–75 p)	88.4	(87–89)	88.1	(86–90)	0.915
TAS, median (25 p–75 p)	2.5	(1.3–2.9)	2.2	(2.1–2.4)	0.830
TOS, mean (SD)	7.8	(0.4)	7.9	(1)	0.800
OSI, median (25 p–75 p)	0.32	(0.28–0.58)	0.36	(0.29–0.41)	0.936

**Table 5:** Comparison of OS markers by polysensitization status in patients with AR.

Oxidative Stress Markers	Allergic Rhinitis				*p*-Value
	Polysensitization = Yes		Polysensitization = No		
	*n* = 22		*n* = 9		
TT (µmol/L), mean (SD)	387	(29)	393	(21)	0.598
NT (µmol/L), mean (SD)	340	(35)	346	(20)	0.662
Disulfide, mean (SD)	24	(6)	24	(3)	0.968
Disulfide/Native Thiol (%), median (25 p–75 p)	6.6	(5.2–8.4)	6.6	(5.9–7.6)	0.632
Disulfide/Total Thiol (%), median (25 p–75 p)	5.8	(4.7–7.2)	5.8	(5.3–6.6)	0.632
NT/TT (%), median (25 p–75 p)	88.4	(86–91)	88.4	(87–89)	0.632
TAS, mean (SD)	2.3	(0.6)	2.2	(0.8)	0.701
TOS, mean (SD)	7.9	(1)	7.9	(0.8)	0.948
OSI, mean (SD)	0.37	(0.12)	0.40	(0.14)	0.609

**Table 6:** Comparison of OS parameters between the AR and control group.

Oxidative Stress Markers	Groups				*p*-Value
	Allergic Rhinitis		Control		
	*n* = 31		*n* = 31		
TT (µmol/L), median (25 p–75 p)	396	(370–410)	425	(388–445)	0.001 **
NT (µmol/L), mean (SD)	342	(31)	380	(40)	0.000 *
Disulfide, mean (SD)	24	(5)	20	(6)	0.012 *
Disulfide/Native Thiol (%), median (25 p–75 p)	6.6	(5.4–8.3)	5.2	(4.4–6.3)	0.001 **
Disulfide/Total Thiol (%), median (25 p–75 p)	5.8	(4.9–7.1)	4.7	(4–5.6)	0.001 **
NT/TT (%), median (25 p–75 p)	88.4	(86–90)	90.5	(89–92)	0.001 **
TAS, median (25 p–75 p)	2.2	(2.1–2.5)	2.3	(2.2–2.6)	0.363
TOS, mean (SD)	7.9	(0.9)	4.8	(1)	<0.0001 *
OSI, median (25 p–75 p)	0.36	(0.3–0.42)	0.21	(0.16–0.25)	<0.0001 **

* Significant difference at <0.05 level according to independent t-test, Means and Standard deviations (SD) are presented ** Significant difference at <0.05 level according to Mann–Whitney U test, Medians are presented and 25 p–75 p are shown in parentheses.

**Table 7:** Correlation analysis findings between laboratory parameters and OS markers.

		TT (µmol/)	NT (µmol/L)	Disulfide	TAS	TOS	OSI
		*n* = 62					
Age (years)	r	0.099	0.101	−0.037	−0.081	−0.052	−0.052
	*p*-Value	0.445	0.434	0.774	0.529	0.688	0.687
BMI (kg/m^2^)	r	0.225	0.190	0.057	0.110	−0.035	−0.072
	*p*-Value	0.079	0.140	0.660	0.394	0.790	0.576
WBC (10^3^/L)	r	0.233	0.195	0.067	−0.202	−0.084	0.000
	*p*-Value	0.069	0.130	0.606	0.115	0.517	0.999
Neutrophil (10^3^/L)	r	0.178	0.177	−0.048	−0.239	0.022	0.205
	*p*-Value	0.167	0.169	0.710	0.062	0.867	0.111
Lymphocyte (10^3^/L)	r	0.092	0.043	0.053	0.111	−0.075	−0.074
	*p*-Value	0.475	0.738	0.683	0.390	0.564	0.566
Monocyte (10^3^/L)	r	−0.191	−0.305	0.343	−0.172	0.378	0.388
	*p*-Value	0.138	0.016 **	0.006 **	0.182	0.003 **	0.002 **
Eosinophil (10^3^/L)	r	0.259	0.290	−0.182	0.149	−0.420	−0.393
	*p*-Value	0.042 **	0.022 **	0.157	0.249	0.001 **	0.002 **
Platelet (10^3^/L)	r	−0.255	−0.278	0.170	−0.128	0.266	0.282
	*p*-Value	0.045 **	0.029 **	0.187	0.323	0.037 **	0.027 **
Hemoglobin (g/dL)	r	0.071	0.052	0.049	−0.182	0.368	0.381
	*p*-Value	0.581	0.691	0.706	0.156	0.003 *	0.002 *
Albumin (g/dL)	r	−0.026	−0.043	0.065	−0.240	0.239	0.377
	*p*-Value	0.840	0.742	0.617	0.063	0.063	0.003 *
CRP (mg/dL)	r	0.092	0.106	−0.124	0.111	−0.187	−0.139
	*p*-Value	0.478	0.413	0.335	0.389	0.146	0.281
Urea (mg/dL)	r	0.020	0.023	−0.038	−0.007	0.213	0.126
	*p*-Value	0.876	0.859	0.768	0.960	0.096	0.328
Creatinine (mg/dL)	r	−0.226	−0.269	0.217	−0.102	0.558	0.440
	*p*-Value	0.078	0.034 *	0.090	0.432	<0.0001 *	0.000 *
ALT (IU/L)	r	0.206	0.186	−0.064	0.137	−0.324	−0.335
	*p*-Value	0.107	0.149	0.623	0.287	0.010 **	0.008 **
AST (IU/L)	r	−0.244	−0.308	0.174	−0.121	0.566	0.495
	*p*-Value	0.056	0.015 **	0.177	0.349	<0.0001 **	<0.0001 **

* Significant correlation coefficient at <0.05 level according Pearson correlation ** Significant correlation coefficient at <0.05 level according Spearman rank correlation.

**Table 8:** Diagnostic performance of OS markers for AR.

	Cut-Off	Sensitivity	Specificity	PPV	NPV	AUC (95% CI)		*p*-Value
TT (µmol/L)	≤415	90	61	70.0	86.4	0.749	(0.622–0.850)	0.000
NT (µmol/L)	≤373.2	90	71	75.7	88.0	0.786	(0.664–0.880)	<0.0001
Disulfide	>20	74	61	65.7	70.4	0.690	(0.560–0.802)	0.005
TAS	≤1.36	16	97	83.3	53.6	0.567	(0.435–0.692)	0.362
TOS	>6.27	97	94	93.7	96.7	0.993	(0.928–1.000)	<0.0001
OSI	>0.28	81	90	89.3	82.4	0.912	(0.812–0.969)	<0.0001

AUC: Area under curve, CI: Confidence interval.

## Data Availability

The raw data supporting the conclusions of this article will be made available by the authors on request.

## Abbreviations

The following abbreviations are used in this manuscript:

AR	Allergic rhinitis
Th2	T helper 2
ROS	Reactive oxygen species
OS	Oxidative stress
TDH	Thiol-disulfide homeostasis
TT	Total thiol
NT	Native thiol
WBC	White blood cell
CRP	C-reactive protein
ALT	Alanine aminotransferase
AST	Aspartate aminotransferase
TAS	Total antioxidant status
TOS	Total oxidant status
MPV	Mean platelet volume
BMI	Body mass index
SAR	Seasonal allergic rhinitis
